# Accurate Measurement of Handwash Quality Using Sensor Armbands: Instrument Validation Study

**DOI:** 10.2196/17001

**Published:** 2020-03-26

**Authors:** Chaofan Wang, Zhanna Sarsenbayeva, Xiuge Chen, Tilman Dingler, Jorge Goncalves, Vassilis Kostakos

**Affiliations:** 1 School of Computing and Information Systems The University of Melbourne Parkville Australia

**Keywords:** hand hygiene, wearable devices, machine learning

## Abstract

**Background:**

Hand hygiene is a crucial and cost-effective method to prevent health care–associated infections, and in 2009, the World Health Organization (WHO) issued guidelines to encourage and standardize hand hygiene procedures. However, a common challenge in health care settings is low adherence, leading to low handwashing quality. Recent advances in machine learning and wearable sensing have made it possible to accurately measure handwashing quality for the purposes of training, feedback, or accreditation.

**Objective:**

We measured the accuracy of a sensor armband (Myo armband) in detecting the steps and duration of the WHO procedures for handwashing and handrubbing.

**Methods:**

We recruited 20 participants (10 females; mean age 26.5 years, SD 3.3). In a semistructured environment, we collected armband data (acceleration, gyroscope, orientation, and surface electromyography data) and video data from each participant during 15 handrub and 15 handwash sessions. We evaluated the detection accuracy for different armband placements, sensor configurations, user-dependent vs user-independent models, and the use of bootstrapping.

**Results:**

Using a single armband, the accuracy was 96% (SD 0.01) for the user-dependent model and 82% (SD 0.08) for the user-independent model. This increased when using two armbands to 97% (SD 0.01) and 91% (SD 0.04), respectively. Performance increased when the armband was placed on the forearm (user dependent: 97%, SD 0.01; and user independent: 91%, SD 0.04) and decreased when placed on the arm (user dependent: 96%, SD 0.01; and user independent: 80%, SD 0.06). In terms of bootstrapping, user-dependent models can achieve more than 80% accuracy after six training sessions and 90% with 16 sessions. Finally, we found that the combination of accelerometer and gyroscope minimizes power consumption and cost while maximizing performance.

**Conclusions:**

A sensor armband can be used to measure hand hygiene quality relatively accurately, in terms of both handwashing and handrubbing. The performance is acceptable using a single armband worn in the upper arm but can substantially improve by placing the armband on the forearm or by using two armbands.

## Introduction

### Background

Health care workers’ (HCWs’) hands play a pivotal role in spreading microorganisms in health care environments and pose a direct clinical threat to patients [1-3]. Health care–associated infections (HAIs), also known as *nosocomial* infections, are the most common causes of morbidity and mortality in hospitals around the world [4]. The average prevalence of HAIs varies from 4.0% to 15.5% in different countries and regions [5-8]. De Angelis et al [9] estimated that patients with HAIs spent approximately 2.5 times more time in hospital and incurred costs that were 2.8 times higher than those for patients free from infection. Moreover, HAIs put a considerable financial burden on the health system: in France alone, nearly 3% of surgical procedures performed in 2010 resulted in infections creating annual costs of €58 million (US $64.5 million) [10], whereas in the United States, the estimated annual costs range from US $28 billion to US $45 billion [11]. Hand hygiene is a simple and cost-effective intervention to prevent HAIs and reduce their transmission [1,12].

In 2009, the World Health Organization (WHO) issued guidelines on Hand Hygiene in Health Care to provide a thorough review of evidence on hand hygiene and recommendations to promote hand hygiene in health care environments [3,13]. The guidelines summarize the key moments for hand hygiene for HCWs as *My 5 Moments For Hand Hygiene*, which have since been the focus of much work on automated technologies [14]. Previous work has reported that the compliance rate of hand hygiene is unacceptably poor [15], with a meta-analysis of 96 empirical studies showing a median compliance rate of 40% among HCWs [16]. A variety of technologies have been developed to increase compliance with the *5 moments for hand hygiene*, such as monitoring staff movement [17], using radio-frequency identification or Bluetooth beacons [14,18,19], and a range of commercial products, such as BioVigil (BioVigil Healthcare Systems, Inc) and SwipeSense (SwipeSense, Inc).

**Figure 1:**
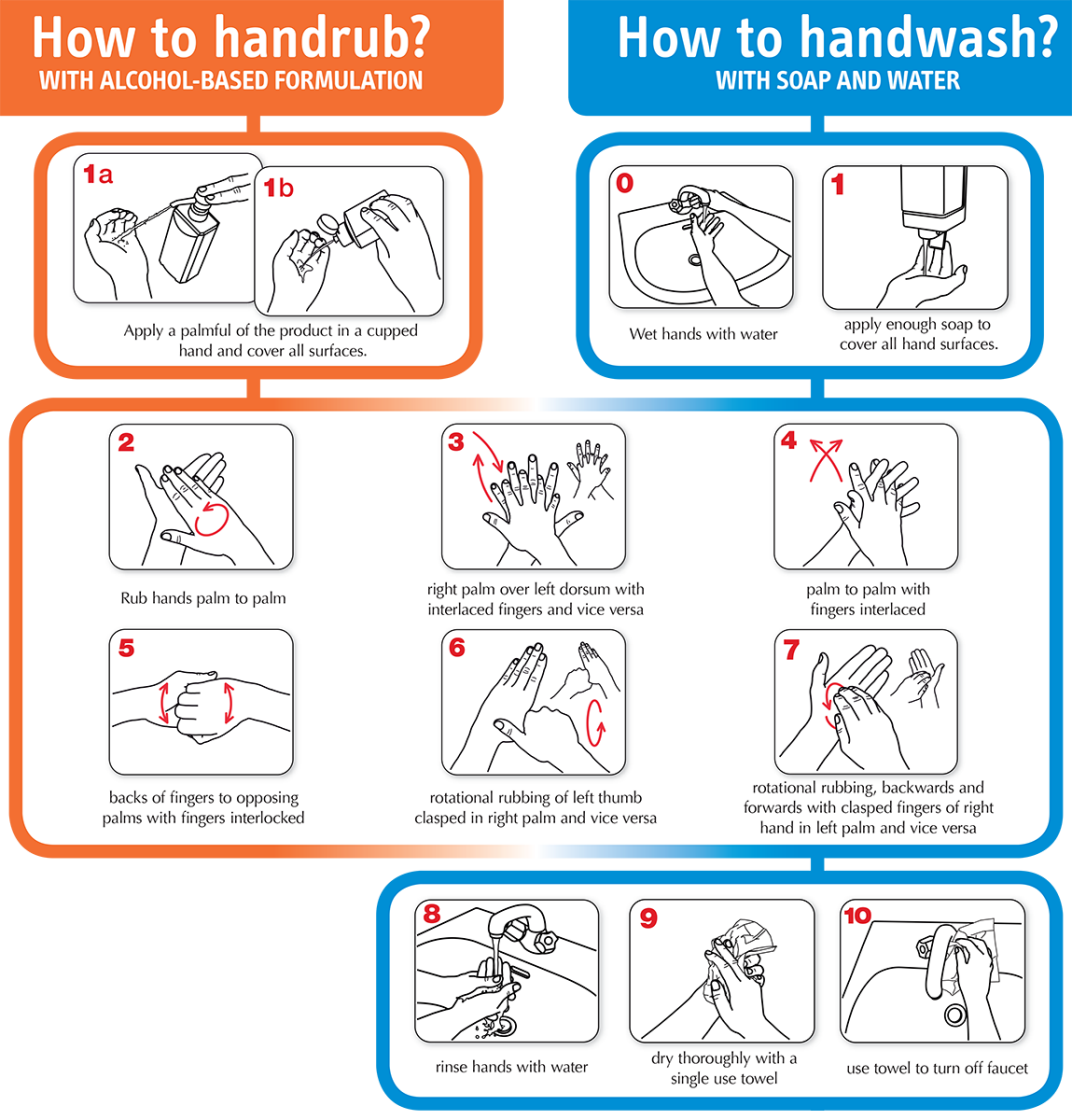
Standard World Health Organization procedures of alcohol-based handrub and handwash with soap and water. Source: World Health Organization. How to Handrub? / How to Handwash? [20]

However, the WHO guidelines also introduced procedures for alcohol-based handrub and handwash with soap and water (shown in Figure 1 [3,20]). These guidelines aim to improve hand hygiene by decreasing colonization with transient flora [12,13]. Especially, alcohol-based handrub is preferred for routine decontamination of hands for all clinical indications, whereas handwash with soap and water is recommended for visibly soiled hands [3]. Different from the compliance rate of hand hygiene, which focuses on whether HCWs perform hand hygiene on time, the compliance with the WHO hand hygiene routines ensures that HCWs achieve adequate coverage of all surfaces of their hands with hand hygiene products [3].

Owing to the technical challenge of monitoring compliance with this set of guidelines, previous work measured compliance with this set of guidelines based on direct observation by trained auditors [21-23]. Yet, compliance with this set of WHO guidelines has been reported to be approximately 8.5% [23]. Crucially, in a large-scale assessment of hand hygiene quality conducted in Singapore, only 72% of HCWs could achieve adequate coverage of all hand surfaces *immediately* after hand hygiene training [24]. These findings suggest that it is important to develop reliable instruments for monitoring the quality of hand hygiene in health care settings by measuring adherence to the WHO handrub and handwash procedures.

Although a wide range of technologies have been developed to measure hand hygiene compliance rate, this is not the case for hand hygiene quality. Most work on quality monitoring has so far relied on cameras. Llorca et al [25] mounted a red green blue camera above a sink to collect hand hygiene practices among HCWs. After performing analysis on color and motion, their system used support vector machine (SVM) to classify six steps of a standard hand hygiene procedure. Similarly, Xia et al [26] collected hand hygiene videos through a red green blue and depth camera. By applying SVM, random forest, and linear discriminant analysis to the collected videos, their system could determine 12 steps of a hand hygiene procedure at the level of a single frame or a single video. A commercially available system is SureWash (Glanta Ltd), which can detect HCWs’ hand motion and provide reminders for the upcoming steps, according to the standard WHO hand hygiene procedure. There has also been some work on hand hygiene–monitoring systems that provide assistance for people with dementia [27]. However, a major concern for camera-based systems to monitor hand hygiene quality is privacy, as those systems inevitably require the installation of cameras in toilets and patient care areas. Furthermore, cameras can only be used in certain environments, and camera-based monitoring systems may not provide actionable data on all hand hygiene events [28]. An additional challenge is that installed cameras cannot be easily moved should the need arise. Especially, cameras are not suitable for monitoring handrub because it tends to happen on the move, although a camera has a fixed field of view. Another concern is that the costs associated with the installation and maintenance of camera systems can be substantial [14].

An alternative to using camera-based systems is to use wearable sensors [29-32]. A common practice in literature is through the use of wristbands [30-32]. Yet, because of hygiene concerns, the WHO recommends removing rings, wristwatches, and bracelets before beginning surgical hand preparation [3,13]. Therefore, it is challenging to use wristbands to monitor hand hygiene procedure compliance, as it can possibly defeat the purpose of hand hygiene. Another limitation with previous studies is the use of Hidden Markov Model (HMM) to classify the steps of the hand hygiene procedure from feature vectors [30] or smooth classification results [29], which assumes HCWs will perform hand hygiene procedures according to the predefined orders. However, once that assumption is relaxed, the performance of these systems dramatically drops, for example, from 85% to 69% [30]. Similarly, previous studies make assumptions about the duration of hand hygiene steps and, for example, collect each step of the hand hygiene procedure individually with, say, a 5-second window [29]. Nonetheless, hand hygiene steps can actually vary rather substantially in duration, from 20 to 30 seconds for handwash to 40 to 60 seconds for handrub [3].

### Objectives

In this paper, we evaluated the accuracy of a sensor armband in measuring compliance with the WHO handrub and handwash guidelines. We used the Myo armband (North Inc) worn on participants’ forearm or arm and evaluated a machine learning classifier that uses eXtreme Gradient Boosting (XGBoost) and E.Divisive [33] to identify each step of the standard WHO handwash or handrub procedures. The analysis evaluated eight different armband placements, six sensor configurations, user-dependent vs user-independent models, and bootstrapping performance.

The contributions of this paper are as follows: (1) unified model—although most previous work focuses on either handrub or handwash [30,34], our work combines these two common activities to create a unified hand hygiene model. Our model can detect each step of the respective WHO procedures. (2) Flexible detection—previous work assumes that HCWs perform hand hygiene according to the expected step order and duration [29,30]. Our model can detect each step individually, regardless of its order, timing, or duration, and can thus provide granular and detailed feedback. (3) Placement recommendations—by analyzing the rich data collected in our study, we can quantify the trade-off between classification accuracy and sensor placement and provide placement recommendations. (4) Sensor recommendations—as the Myo armband consists of a nine-axis inertial measurement unit (IMU) and eight electromyographic electrodes, we were able to collect acceleration, gyroscope, orientation, and surface electromyography (sEMG) data simultaneously [35]. By evaluating different sensor data combinations, we were able to quantify the trade-off between accuracy and sensor types and recommend sensors for future hand hygiene–monitoring systems.

## Methods

### Task and Hardware Specification

We adopted the procedures for alcohol-based handrub and handwash with soap and water (Figure 1 [20]) recommended by the WHO guidelines on Hand Hygiene in Health Care [3]. The procedure contains seven steps for handrub and 11 steps for handwash (shown in Figure 1 [20]). Some of the steps are repeated for each hand. The guidelines claim that the total duration of the handrub routine is 20 to 30 seconds on average, whereas handwash lasts for 40 to 60 seconds.

We summarized all the steps for both procedures in Table 1. For handwash, we decided to exclude step 0 (wet hands with water) and step 1 (apply hand hygiene product), as the position of faucets and hand hygiene dispensers can vary across different lavatories. Furthermore, as previous work has noted [34], hand dominance is not crucial to hand hygiene detection because arm movements are symmetric. For this reason, our analysis does not consider the hand dominance effect.

**Table 1:** Steps of the World Health Organization hand hygiene procedures.

Step #	Steps of the World Health Organization hand hygiene procedures	
	Alcohol-based handrub	Handwash with soap and water
0	N/A^a^	N/A
1	Apply hand hygiene products	N/A
2	Rub palm	Rub palm
3 (R^b^)	Rub dorsum (R)	Rub dorsum (R)
3 (L^c^)	Rub dorsum (L)	Rub dorsum (L)
4	Interlock fingers	Interlock fingers
5 (R)	Twist knuckles (R)	Twist knuckles (R)
5 (L)	Twist knuckles (L)	Twist knuckles (L)
6 (R)	Rub thumb (R)	Rub thumb (R)
6 (L)	Rub thumb (L)	Rub thumb (L)
7 (R)	Scrub fingertip (R)	Scrub fingertip (R)
7 (L)	Scrub fingertip (L)	Scrub fingertip (L)
8	N/A	Rinse hands
9	N/A	Dry hands with towel
10	N/A	Turn off faucet with towel

^a^Not available.

^b^R: right.

^c^L: left.

The hardware for this study was the Myo armband, which contains a nine-axis IMU sensor and eight electromyographic electrodes. Through the nine-axis IMU sensor, the armband captures acceleration, gyroscope, and orientation data at a sample rate of 50 Hz. Using the eight surface electromyographic electrodes, it can also collect sEMG signals to measure users’ muscular activity at a frequency of 200 Hz. Physiological data were transmitted to a receiver (in our case, a laptop) via the Bluetooth Low Energy protocol. Data from multiple armbands were simultaneously collected using the Myo SDK [35] and modified myo-python library [36] and synchronized using the algorithm proposed by Wang et al [37].

### Experiment Design

Our experimental design had four independent variables: hygiene mode (handwash vs handrub), instruction mode (video vs poster), armband placement (above elbow vs below elbow), and hand (left vs right hand). We measured the time taken to complete each step of each hygiene procedure and the errors made by participants when washing their hands. We also measured the accuracy of the classifier in detecting each step of the procedure. The experiment followed a within-subjects design, and all participants completed all conditions in a counterbalanced manner. The University of Melbourne’s Engineering Human Ethics Advisory Group approved the study.

We recruited 20 participants through our university’s mailing lists and snowball recruitment with an equal number of women and men. All participants were students or staff in our university, and their ages ranged between 22 and 33 years (mean 26.5 years, SD 3.31). The majority of participants (18/20, 90%) had not received formal training in hand hygiene in the last 3 years and were not familiar with the formal hand hygiene procedures. A total of 95% (19/20) of participants reported using their right hand as their dominant hand and no ambidextrous participants.

**Figure 2:**
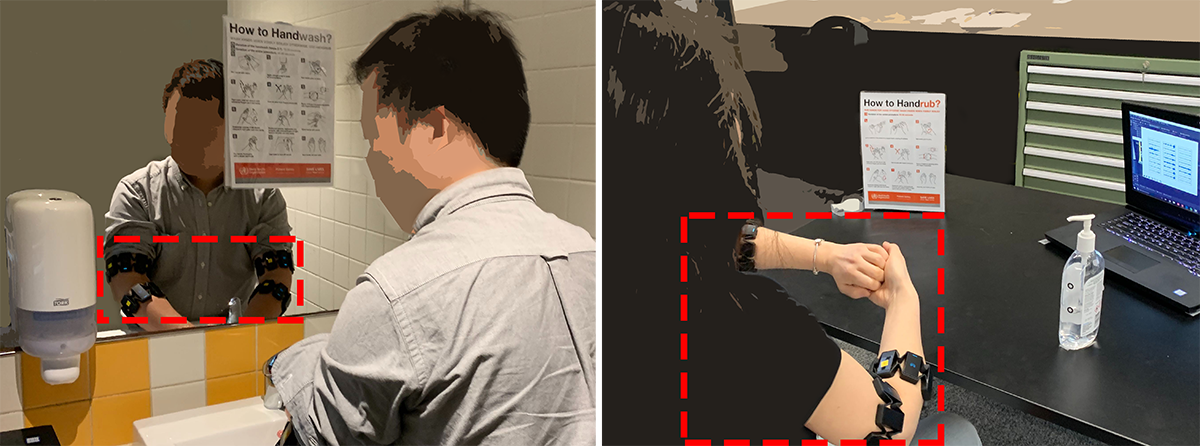
Handwash with soap and water (left) and alcohol-based handrub (right).

On arrival at our laboratory, we briefed participants on the purpose of the study and obtained their written consent agreeing to participate in our experiment. Then, we asked our participants to wear four Myo armbands on their forearms and arms, as shown in Figure 2. Thus, each participant had an armband in their upper and lower left arm and upper and lower right arm.

We subsequently provided training to our participants. We instructed them on how to perform the handwash and handrub procedures. To achieve this, we first explained to them the procedure steps using an instructional poster (Multimedia Appendices 1 and 2) [3]. We then asked participants to watch an instructional video five times while they perform the procedure. We ran this process twice: once for handrub [38] and once for handwash with soap and water [39] in a counterbalanced manner.

**Figure 3:**
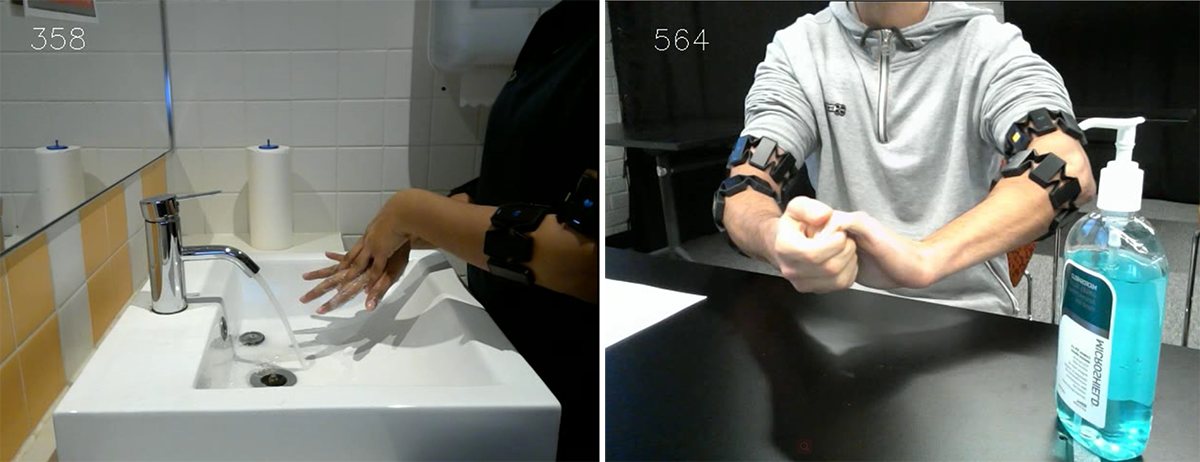
Screenshots of the video records of handwash with soap and water (left) and alcohol-based handrub (right). The frame number (top left) is used to synchronize video data to the armband data.

After training, we initiated the main experimental task, during which we collected data from the four armbands that participants wore, and we also videotaped participants’ hands as a way of capturing the ground truth (Figure 3). Each participant performed 30 sessions: five handwash sessions where they could follow the instructional video, 10 handwash sessions accompanied with the instructional poster, five handrub sessions with the instructional video, and 10 handrub sessions with the instructional poster. We counter balanced the order of the conditions to avoid order effects. Handrub was performed in our laboratory, and handwash was performed at an adjoining lavatory (Figure 2). The overall duration for each participant was approximately 70 min. Each participant was rewarded with an AUD $20 (US $13.4) gift card regardless of their performance.

### Data Analysis

Our first step was to annotate the video recordings manually: we visually inspected all recordings (493 min in total) and annotated each video frame as belonging to one of the 14 handwash steps. All the video recordings were inspected by two data annotators (annotator 1: 16 participants and annotator 2: 4 participants), and annotator 1 checked the annotations made by annotator 2. The interrater reliability was 1.0 for those samples with two annotators. In total, we made 7180 manual annotations, which represented our ground truth. Using these annotations, along with the frame numbers of the video recordings, we then annotated the armband data with the relevant hand hygiene step label.

Next, we were able to measure the accuracy of the algorithm for classifying the steps of the hand hygiene procedures, which operates as follows: (1) preprocessing, (2) feature extraction, (3) classification, (4) postprocessing, and (5) evaluation.

#### Preprocessing

Owing to the different frequencies of IMU (50 Hz) and sEMG (200 Hz) data, we downsampled both types of sensor data to 25 Hz. We then normalized the downsampled data by applying z-score normalization and applied a sliding window procedure with a 0.2-second time window with 75% overlap. We also measured the classification accuracy for different sliding window parameters, ranging from 0.1 seconds to 0.4 seconds and from 50% overlapping to 75% overlapping, and found the sliding window with a 0.2-second time window and 75% overlap gave the best classification accuracy.

#### Feature Extraction

Features extracted from the time domain and the frequency domain are widely used in activity recognition tasks. By summarizing previous work [40-43], we calculated all the selected features for the collected IMU and sEMG data. Table 2 shows the extracted features, and Multimedia Appendix 3 provides the details of features. The dimensionality of the feature vector can vary on different configurations (eg, the number of armbands and sensors). Then, to minimize the required computational power and computation time for the activity recognition task, we used boosting algorithms to select discriminative features [44]. In particular, we used XGBoost [45] to choose the most discriminative subset (top 100) of features according to occurrences of the features in splits; hence, the final feature vector had a dimensionality of 100.

**Table 2:** Features are extracted from acceleration, gyroscope, orientation, and surface electromyography data.

Features	Study authors
CSD, peak (positive), peak (negative), RMS	McIntosh et al [40]
ACAbsArea, ACAbsCV, ACAbsMean, ACEntropy, ACIQR, ACKur, ACQ1, ACQ3, ACRange, ACSkew, ACVar, DCArea, DCMean, DCPostureDist, DCTotalMean	Munguia Tapia et al [41]
|AL|, |∆AL|, |∆AR|, |∆AR|, |∆MAV|, AJ, AL, AR, RAJ, RMAV, SAJ, SDAL, SDAR, SRAJ	Xie et al [42]
meanPKT, meanPSD, medainS, medianPKT, medianPSD, stdPKT, stdPSD, stdS	Zhang et al [43]

#### Classification

We fed the generated feature vectors and the ground truth labels to XGBoost to classify the steps of the WHO hand hygiene procedures shown in Table 1 because XGBoost is also widely used in human activity recognition tasks [46,47]. Other techniques were considered and tested during the pilot study, including random forest and SVM, but they performed worse than XGBoost as same as previous studies [46,47]. XGBoost has been shown to have several other advantages, including high efficiency, low computational cost, supporting parallelization, and robustness to overfitting [47]. For these reasons, we focused on applying XGBoost to recognize the steps of hand hygiene procedures.

#### Postprocessing

A final step of the data analysis pipeline in our study was smoothing the stream of predictions to remove classification errors because switching from one gesture to another gesture several times per second was not realistic. Instead of HMM used in previous studies, we smoothed the prediction stream through a combination of E.Divisive [33] and majority vote. E.Divisive is a nonparametric multiple change point analysis approach based on hierarchical clustering, which can detect distributional changes from a sequence of data [33]. By fitting the prediction stream into E.Divisive using the ecp library [48], we could estimate the location of the change points and use the change points to segment the prediction stream. Finally, the class of each segment was determined by a majority vote over the predictions in the segment.

#### Evaluation

We measured the accuracy of both a user-independent model (one model to classify data for all participants) and user-dependent models (one model tuned to each participant). To measure the accuracy of models, we used leave-one-session-out (LOSO) cross-validation for the user-dependent model and leave-one-participant-out (LOPO) cross-validation for the user-independent models, as suggested in the literature [30].

In LOSO cross-validation, we considered 29 hand hygiene sessions from one participant for training and tested the model on the holdout session. We performed this protocol 30 times per participant (thus, each session became the holdout session once) and calculated the average accuracy per participant. We then repeated this procedure for each participant independently.

To evaluate the user-independent model, we used 19 participants’ hand hygiene data to train the model and tested the model on the remaining participant. We repeated the cross-validation for every participant in our database and then averaged the results across the 20 runs.

## Results

Throughout our results, we make an explicit distinction between the user-independent and the user-dependent models. This is because of the practical implications of choosing one approach over the other. A user-independent model *works for all users* and can be used by an HCW without prior training. A user-dependent model needs to be trained and tuned to each HCW individually. The benefit of the former is that it does not require further training (also known as bootstrapping), whereas the latter attains improved performance.

### Participant Performance

We first considered the participants’ performance in terms of hand hygiene quality, and, specifically, we were interested in measuring any variations to their performance. As we gave identical and considerable training to each participant, we expect that their hand hygiene quality is high and consistent. By analyzing our annotations of the recorded videos, we can achieve the following:

- Measure the exact duration of hand hygiene sessions and steps in those sessions and how they vary across conditions;
- Identify the order in which participants actually performed hand hygiene and whether they missed or skipped steps or performed steps incorrectly; and
- Investigate the presence of possible learning or fatigue effects in the study.

#### Time to Complete Hand Hygiene

**Figure 4:**
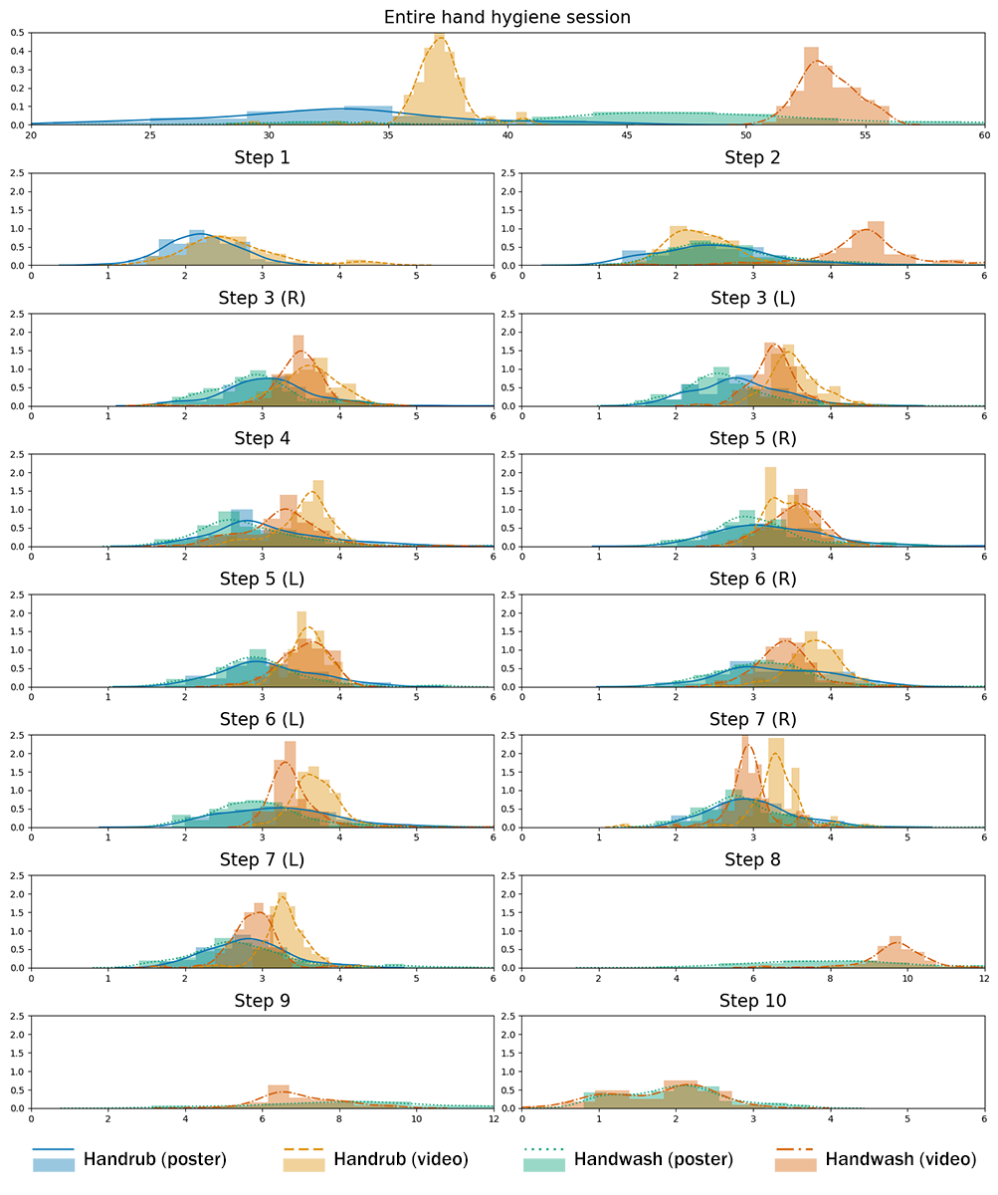
Duration of hand hygiene sessions and inner steps. x-axis: duration (seconds); y-axis: density. L: left; R: right.

By summarizing the duration of hand hygiene sessions in the top of Figure 4, we observe that duration varies considerably across different conditions as follows: handwash with the instructional video (mean 53.4 seconds, SD 1.1) or poster (mean 47.9 seconds, SD 8.3) and handrub with the video (mean 37.1 seconds, SD 1.4) or poster (mean 32.6 seconds, SD 5.3). We observed this effect for overall timings and the duration of each specific step. A one-way analysis of variance (ANOVA) showed a statistically significant effect of the different conditions on the duration of hand hygiene sessions (*F*_3,596_=402.83; *P*<.001). The post hoc Tukey’s honestly significant difference test indicated that the duration of the video sessions is longer than that of the poster sessions, and the duration of the handwash sessions is longer than that of the handrub sessions.

Furthermore, we observed that participants’ time hygiene varied considerably more (higher standard deviation) in the poster conditions as opposed to the corresponding video conditions. This is not surprising, as the video imposes a certain pace on participants, whereas in the poster condition, participants are free to set their own pace.

#### Step Sequence Accuracy

**Figure 5:**
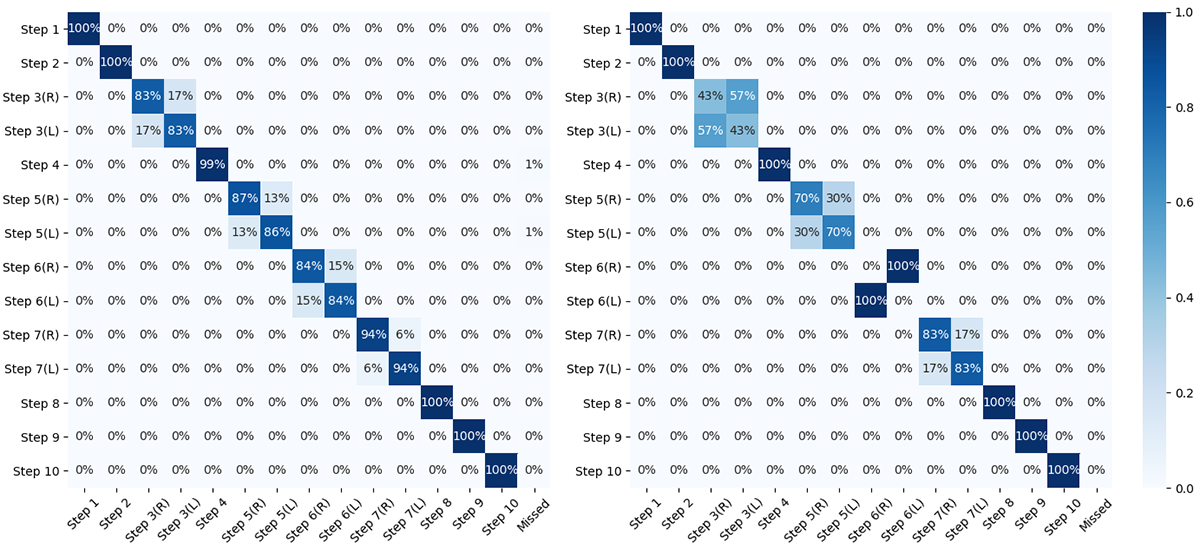
Confusion matrix showing the order of steps in hand hygiene sessions: all participants (left) and participant 8 (right). x-axis: performed step; y-axis: excepted step. L: left; R: right.

By observing the recorded hand hygiene sessions, we noticed that participants did deviate from the defined procedure and had an overall accuracy of 91% (SD 0.16) in terms of complying with the protocol. As shown in Figure 5, we calculated the average participant accuracy as a measure of their compliance for each of the protocol steps. Participants tended to swap those steps that need to be repeated symmetrically with different leading hands, including step 3(R)/3(L), step 5(R)/5(L), step 6(R)/6(L), and step 7(R)/7(L). We also observed that on some occasions, participants missed a step (denoted as rightmost column *Missed* in Figure 5).

On the right of Figure 5, we show exemplary data collected from participant 8, who swapped the order of steps 6(R) and 6(L). We noted that this participant was right-handed. Overall, we observe that participants are able to follow the standard WHO hand hygiene procedures to a great extent, although participants may accidentally miss some steps or swap some symmetrical steps. We highlight that although these errors were made by participants, they do not affect the accuracy of our classifier. This is because we manually inspected and labeled our video recordings, and therefore, any missed or swapped steps are assigned the correct ground truth label.

#### Learning and Fatigue Effects

**Figure 6:**
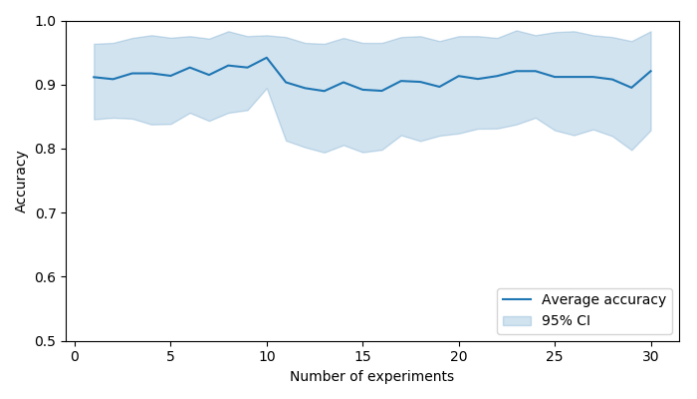
Average participant accuracy, according to the number of completed hand hygiene sessions.

To quantify the existence of any potential learning or fatigue effects, we calculated participants’ average accuracy after having completed one procedure, two, three, and so on until 30. The results are shown in Figure 6. The average accuracy remained at around 90% across the range of 1 to 30 completed sessions. A one-way ANOVA showed no statistically significant effect of the number of experiments on the average accuracy (*F*_29,570_=0.10; *P*>.99). This indicates that participants did not have a significant increase or decline in performance, which would be suggestive of learning or fatigue, respectively.

### Armband Performance

#### Placement of Armbands

**Figure 7:**
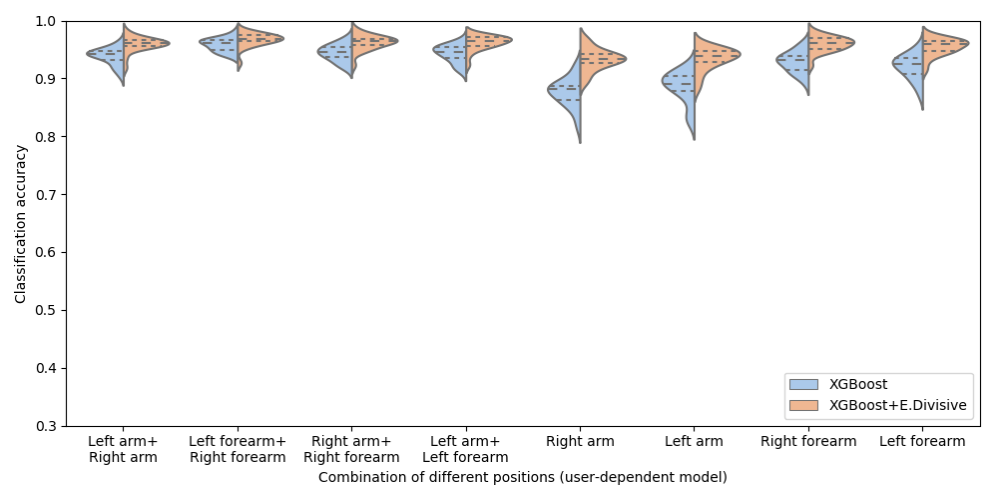
Classification accuracy of different combinations of armband positions: user-dependent models.

**Figure 8:**
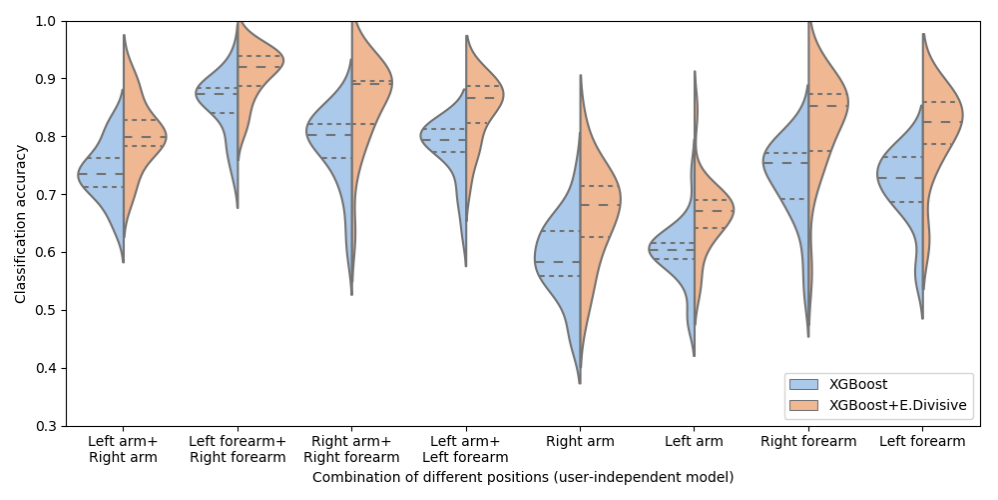
Classification accuracy of different combinations of armband positions: user-independent model.

To investigate the performance of the armband, we calculated the accuracy for eight distinct combinations of armband positions: four combinations of two armbands (left arm+right arm, left forearm+right forearm, right arm+right forearm, and left arm+left forearm) and four positions of one armband (right arm, left arm, right forearm, and left forearm). We separately calculated the average accuracy for the classification results (XGBoost) and the smoothed classification results (XGBoost+E.Divisive). At this point, the results are derived using a fusion of all available sensor data (acceleration, gyroscope, orientation, and sEMG) and are shown in Figure 7 (user-dependent models) and Figure 8 (user-independent model). As noted earlier, we used LOSO cross-validation for the user-dependent models and LOPO cross-validation for the user-independent model.

For the user-dependent model, Figure 7 shows the best performance was achieved when using the data from both left forearm and right forearm (XGBoost: mean 95.9%, SD 0.01; and XGBoost+E.Divisive: mean 96.9%, SD 0.01). For situations where only one armband is desirable or available, the best model uses data from the right forearm (XGBoost: mean 93.0%, SD 0.02; and XGBoost+E.Divisive: mean 96.1%, SD 0.01).

Similar result trends were observed in the user-independent model as well, although the user-independent model lags in performance as expected. In Figure 8, the model using the data from both the left forearm and right forearm (XGBoost: mean 85.5%, SD 0.05; and XGBoost+E.Divisive: mean 90.9%, SD 0.04) outperformed other placement combinations. When considering only one armband, the highest classification accuracy was also achieved by the model using the data from the right forearm (XGBoost: mean 72.6%, SD 0.07; and XGBoost+E.Divisive: mean 82.4%, SD 0.08).

Although we observed that the overall classification accuracy of the user-independent model is lower than that of the user-dependent models, we found that the user-independent model using the data from two armbands is able to achieve more than 80% accuracy.

**Figure 9:**
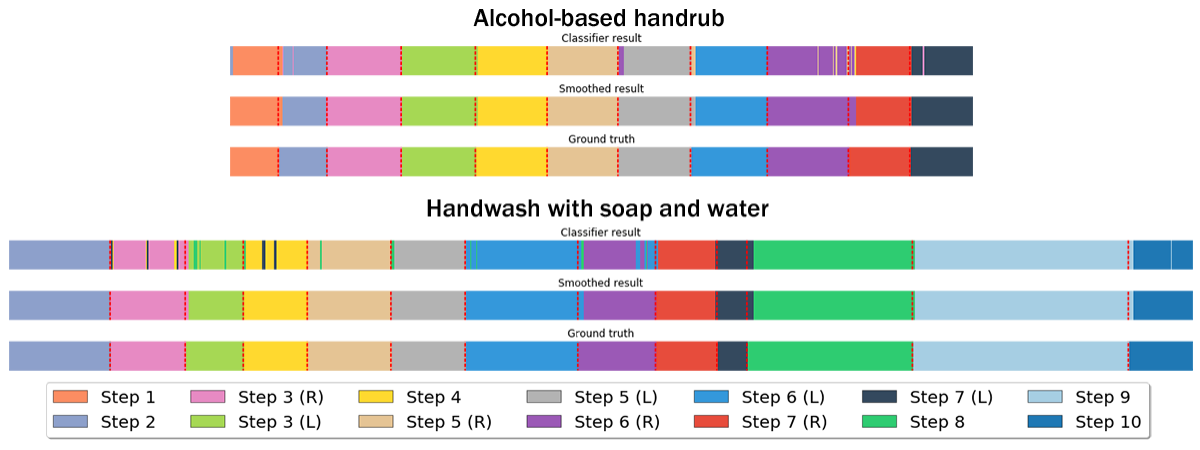
Visualization of how classification and smoothing work for handrub (top) and handwash (bottom). The steps are in accordance with Table 1.

There were several similarities between the results for the user-dependent models and the user-independent model, lending higher robustness to our results. We observed that the classification accuracy increases after smoothing in all cases, shown in Figures 7 and 8 with a two-way ANOVA showing a significant improvement with smoothing (user-dependent model: *F*_1,311_=197.81; *P*<.001; and user-independent model: *F*_1,311_=111.40; *P*<.001). Figure 9 illustrates how the E.Divisive smoothing algorithm improves the data quality by reducing classification errors. Figure 9 also demonstrates how the classifier can detect steps that are out of order (steps 6(R) and 6(L) were performed in reverse order by the participant), deal with missed steps (step 5(L) was skipped), and detect steps with varying duration. Furthermore, Multimedia Appendices 4 and 5 show the confusion matrices of recognition rates from user-dependent models and user-independent models, respectively.

#### Bootstrapping User-Dependent Models

The results have shown that in scenarios where only one armband is placed on the arm, the performance of the user-independent model drops to below 70% (Figure 8). Considering the low accuracy associated with user-independent models, it would be desirable to use a user-dependent model that can achieve higher classification accuracy of about 95% (Figure 7). However, this requires training data from the individual user, and therefore, the model cannot perform well immediately [49]. This can pose an additional burden on HCWs who need to train the model before it performs well. For this reason, we quantified the amount of training data that is required to achieve a reasonable classification accuracy.

**Figure 10:**
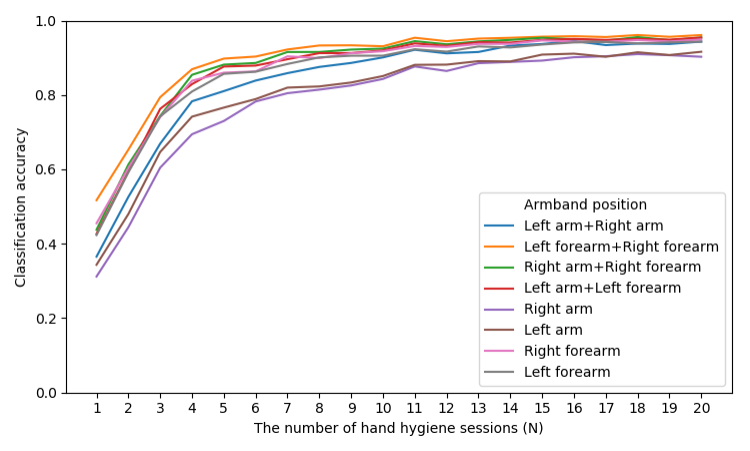
Classification accuracy of user-dependent models using N hand hygiene sessions as the training set.

We did this by randomizing the order of both 15 handrub and 15 handwash sessions for each participant, pick 1, 2, ..., *N* hand hygiene sessions as the training set, and test the model on the remaining hand hygiene sessions for that participant. We repeated this process for every participant and calculated the average classification accuracy for each *N.* The results are shown in Figure 10 and illustrate that classification accuracy rapidly increases as the number of training sessions grows from 1 to 6. With six hand hygiene sessions as the training set, the models with one armband placed on participants’ arm achieve around 80% accuracy. After 16 hand hygiene sessions, the accuracy of the models solely based on the arm is higher than 90%.

#### Sensor Importance

It is crucial to minimize the interruptions of HCWs when using wearable technology such as the armbands in our study, for example, to recharge the armband. Therefore, one approach is to minimize the number of sensors on the armband and therefore increase the battery lifetime but reduce the classification accuracy. For this reason, we quantified the trade-off between the power consumption and classification accuracy of the wearable sensors. In addition, one of the major concerns of electronic hand hygiene–monitoring systems is the cost associated with the wearable sensors and corresponding receivers, and therefore, reducing the sensors can reduce the associated costs. Furthermore, another concern is HCWs’ acceptance and compliance with hospital regulations (eg, not wearing wristbands or watches), and thus, we expected to minimize the number of sensors and the size of sensor armbands to reduce their impediments and increase HCWs’ acceptance.

**Figure 11:**
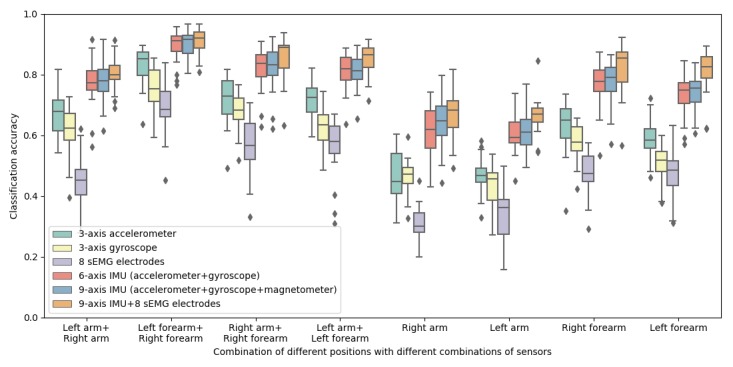
Classification accuracy of the user-independent model for different combinations of sensors and different placement. IMU: inertial measurement unit; sEMG: surface electromyography.

To quantify this energy vs performance trade-off, we fed the user-independent model with the data from different combinations of sensors in line with the widely used wearable devices. Specifically, we examined the performance of the user-independent model with six different combinations of sensors through LOPO cross-validation. The six combinations are as follows: three-axis accelerometer, three-axis gyroscope, eight sEMG electrodes, six-axis IMU (accelerometer and gyroscope), nine-axis IMU (accelerometer, gyroscope, and magnetometer), and nine-axis IMU + eight sEMG electrodes. Effectively, these are all subsets of the full dataset we collected, and that is why this analysis is possible. The classification accuracy when using different sensor combinations, with different sensor placement, is shown in Figure 11.

Figure 11 shows that, in general, performance suffers when the available data are only a three-axis accelerometer, or a three-axis gyroscope, or eight sEMG electrodes. This finding holds across all armband placements for both one- and two-armband scenarios. The results also showed that if only one sensor is available, then a three-axis accelerometer would be preferable, as it outperforms the others. However, we also observe in Figure 11 a substantial performance gain when a six-axis accelerometer is used—effectively when an accelerometer and gyroscope are combined. Further adding a magnetometer (ie, nine-axis IMU), and subsequently adding the sEMG data, provides mostly marginal performance gains.

## Discussion

### Flexible Detection

To mimic the real-life scenarios in health care settings, we did not place restrictions on the completion time in our experiments. As a result, we observed that the duration of hand hygiene performance varied considerably when the sessions were accompanied by the instructional poster. Interestingly, performing hand hygiene with posters is common practice in hospitals, and Multimedia Appendices 1 and 2 have been widely used as an approach to promote hand hygiene [50]. In addition, the suggested duration of handwash and handrub from the WHO also varies from 20 to 30 seconds and from 40 to 60 seconds, respectively [3]. Hence, to detect the steps of hand hygiene procedures in realistic scenarios, the system should be adaptive to the varying duration.

Our study also shows that participants did deviate from the defined procedure, especially for the steps that need to be repeated symmetrically with different leading hands. Meanwhile, some participants accidentally missed some of the steps, which is more prevalent in health care settings. For example, Tschudin-Sutter et al [23] reported a compliance rate of only 8.5% for completing all steps of the WHO hand hygiene procedures. Furthermore, the researchers do not have a consensus on the correct order of the hand hygiene procedure steps [51]. Pires et al [51] suggested that HCWs should rub the fingertips first to reduce the probability of contamination. Hence, to monitor the quality of hand hygiene, the system should provide the flexibility of detecting the individual steps within the hand hygiene procedures.

Previous studies adopted HMM to classify the steps of the hand hygiene procedure from feature vectors or smooth classification results [29,30], which assumes that HCWs will perform hand hygiene within a specific time frame and with a predefined sequence. Instead, we smoothed the prediction results through a combination of E.Divisive and majority vote. The results indicated that the classification accuracy increases after smoothing (user-dependent model: *F*_1,311_=197.81; *P*<.001; and user-independent model: *F*_1,311_=111.40; *P*<.001; see Figures 7 and 8). Our approach can deliver flexibility for detecting hand hygiene in real-life scenarios without assuming that the HCWs follow a predefined sequence within a specific time limit.

### Placement Recommendations

To investigate the relationship between the performance of our proposed models and the placement of the armbands, we tested the models with eight placement combinations. For a scenario with two armbands, the model using the data from both the left forearm and the right forearm exhibits the highest classification accuracy (user-dependent model: 96.9% and user-independent model: 90.9%). When using the data from both the right arm and the left arm, the performance decreases to 93.8% and 80%, respectively. However, it can be argued that placing the armbands on the forearm is less hygienic that placing them on the arm.

When only one armband is available, the performance of the user-dependent model still reaches more than 93.3% accuracy regardless of the position of the device (eg, on the lower or upper arm). However, for the user-independent models, the performance drops below 70% when the armband is located on the upper arm (80% accuracy for the lower arm). Thus, a user-dependent model is preferred when only using the data from one armband.

Furthermore, to reduce the costs of data collection, we measured the amount of training data that is required to achieve a reasonable classification accuracy. With six hand hygiene sessions as the training set, the models in the one armband scenario placed on participants’ arm achieve around 80% accuracy for the user-dependent model. After 16 hand hygiene sessions, the accuracy of the models is higher than 90%. However, these models need manually annotated personal training data, which might require considerable costs associated with personnel time and resources.

Overall, for the scenarios allowing HCWs to wear armbands on their forearms, the user-independent models can achieve acceptable classification accuracy (one armband: more than 80% and two armbands: 90.9%). When the hygiene protocol enforces restrictions on HCWs’ forearms (eg, the armband is not allowed on the forearm), the user-independent model needs data from both the left arm and right arm because the performance of the user-independent models using one armband is lower than 70%. Owing to this, user-dependent models are necessary and should be trained by six annotated hand hygiene sessions at least to achieve reasonable classification accuracy.

### Sensor Recommendations

To minimize the interruptions caused by the wearable sensors (eg, recharge the armbands), one can aim to reduce the number of sensors on the armband so as to increase the battery lifetime. Therefore, we quantified the trade-off between the number of wearable sensors and the corresponding classification accuracy. Our results showed that if only one sensor is available, then a three-axis accelerometer would be preferable, as it outperforms the other sensors. After combining an accelerometer and a gyroscope, the model shows a substantial increase. Further adding a magnetometer (ie*,* nine-axis IMU), and subsequently adding the sEMG data, provides marginal performance gains at the cost of substantial power consumption. Other studies also showed that after adding sEMG data, the performance of user-independent models does not exhibit a considerable increase [52,53].

These results point to two sensor combinations that appear to minimize power consumption and cost while maximizing performance. For a single-sensor configuration, a three-axis accelerometer is preferable. For multisensor configurations, a six-axis IMU (ie*,* accelerometer+gyroscope) is the parsimonious combination of choice. We also observed that for single-armband scenarios, the classification accuracy with single-sensor data is largely below 60%, and therefore, a multisensor configuration is appropriate where only one armband is available.

### Feedback

One of the key steps for medical students and HCWs to acquire the clinical techniques is providing feedback on their performance in given activities [54]. Before receiving feedback regarding their performance of hand hygiene, the first step should be to reveal the gap between the optimal and actual performance.

In practice, the duration of a hand hygiene procedure is considered as the key indicator of quality [22,55]. Meantime, as mentioned by Arias et al [21] and Tschudin-Sutter et al [23], the noncompliance with all steps of hand hygiene procedures results in failure to cover all skin surfaces; hence, an automated monitoring approach should also provide information about the sequence of hand hygiene steps actually followed. Owing to the flexibility of our system, we can detect a hand hygiene procedure with different duration and orders. Therefore, we can provide feedback to medical students and HCWs on the duration of hand hygiene procedure (eg, which steps are performed, how much time they spent on the specific steps, and whether they should prolong the duration of the specific step) and the sequence of hand hygiene procedures (eg, whether there is any missed step and whether performed steps are in the correct order).

Through instructional applications, such feedback can be based on the individual performance of hand hygiene so that the trainees can receive more detailed and personalized instructions during the training period. This approach can be used to investigate the performance of HCWs’ daily hand hygiene events. HCWs can further improve their hand hygiene techniques through timely and periodic feedback. Similarly, administrators can also have summarized information to help them quantify hand hygiene quality.

### Limitations

In this study, we recruited participants through our university’s mailing list and collected data in a controlled laboratory setting and did not conduct a field study in health care settings. However, it was necessary to conduct a laboratory study first to provide a semicontrolled setting for instrument validation. We also provided substantial training to participants, and we did observe their performance to be higher than 90%, suggesting that our training was effective in ensuring they follow the WHO guidelines during the study.

Another limitation is that we did not measure the effect of handedness on classifier performance because of the small number of left-handed participants (1/20, 5% participants). However, as mentioned by Galluzzi et al [34], hand dominance is not crucial to hand hygiene detection because of symmetric arm movements.

In addition, we limited our approach to measuring the performance of hand hygiene quality to Myo armbands, which have officially ended as of October 2018. Nevertheless, we argue that a similar approach should be applicable with other wearable devices that bear IMU + electromyography sensors, so long as our proposed analysis is adopted.

### Conclusions

In this paper, we evaluated the feasibility of using sensor armbands (Myo armband) to assess the HCWs’ compliance with the WHO hand hygiene guidelines, which are considered as the proxy measures of the quality of hand hygiene. Our results showed the classification performance of 97% average accuracy for the user-dependent model and 91% average accuracy for the user-independent model. In addition, by investigating the performance of individual models with different sizes of training data, we found that training the user-dependent model with six annotated hand hygiene events can provide more than 80% accuracy with the data from one armband placed on the users’ upper arms. We also investigated the performance of different sensor combinations and found that the combination of an accelerometer and a gyroscope achieves the balance between the classification performance, power consumption, and cost. Our findings contribute to building mechanisms to quantify the quality of hand hygiene procedures using a sensor armband.

## Appendix

Multimedia Appendix 1 Standard World Health Organization procedure of handwash with soap and water. Source: WHO Guidelines on Hand Hygiene in Health Care: First Global Patient Safety Challenge Clean Care is Safer Care [3]

Multimedia Appendix 2 Standard World Health Organization procedure of alcohol-based handrub. Source: WHO Guidelines on Hand Hygiene in Health Care: First Global Patient Safety Challenge Clean Care is Safer Care [3]

Multimedia Appendix 3 Description of the selected features.

Multimedia Appendix 4 Confusion matrix showing the recognition rates of user-dependent models according to different placement combinations.

Multimedia Appendix 5 Confusion matrix showing the recognition rates of user-independent models according to different placement combinations.

## Abbreviations

ANOVA: analysis of variance
HAI: health care–associated infection
HCWs: health care workers
HMM: Hidden Markov Model
IMU: inertial measurement unit
LOPO: leave-one-participant-out
LOSO: leave-one-session-out
sEMG: surface electromyography
SVM: support vector machine
WHO: World Health Organization
XGBoost: eXtreme Gradient Boosting
