# Supplementary material for: Accurate Measurement of Handwash Quality Using Sensor Armbands: Instrument Validation Study
Source: JMIR Mhealth Uhealth. 2020 Mar 26;8(3):e17001. doi: 10.2196/17001 (PMC7146248; doi:10.2196/17001)
Supplement: Multimedia Appendix 2 [file mhealth_v8i3e17001_app2.pdf]

How to Handrub?

RUB HANDS FOR HAND HYGIENE! WASH HANDS WHEN VISIBLY SOILED

Duration of the entire procedure: 20-30 seconds

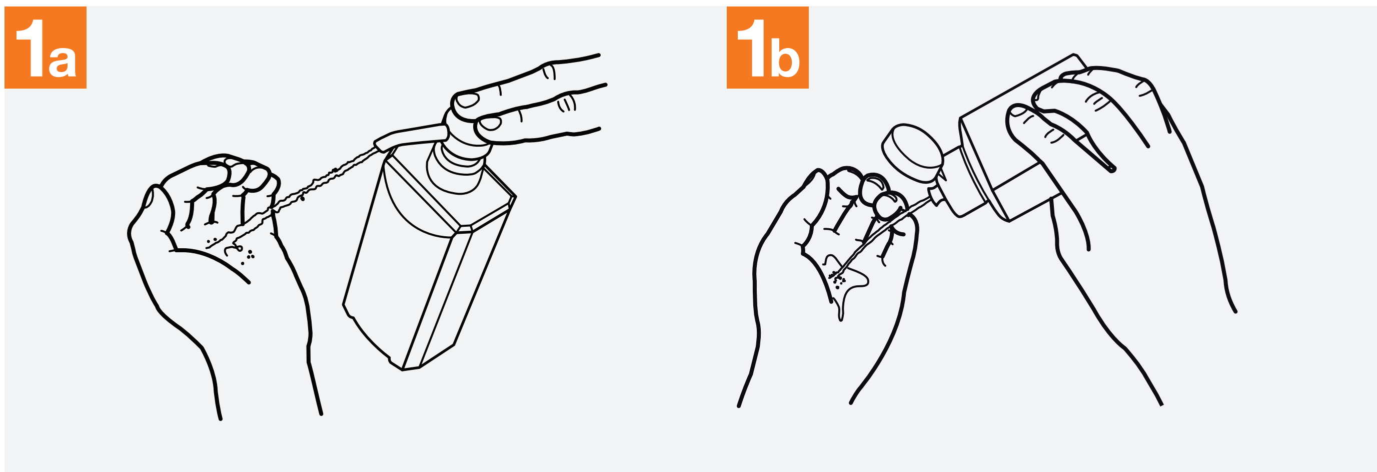

Apply a palmful of the product in a cupped hand, covering all surfaces;

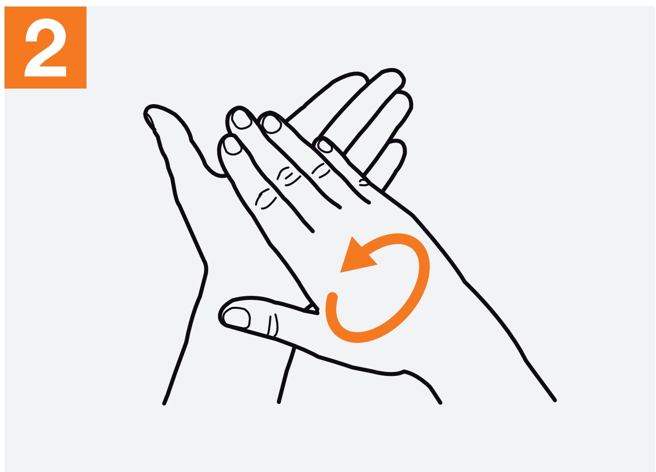

Rub hands palm to palm;

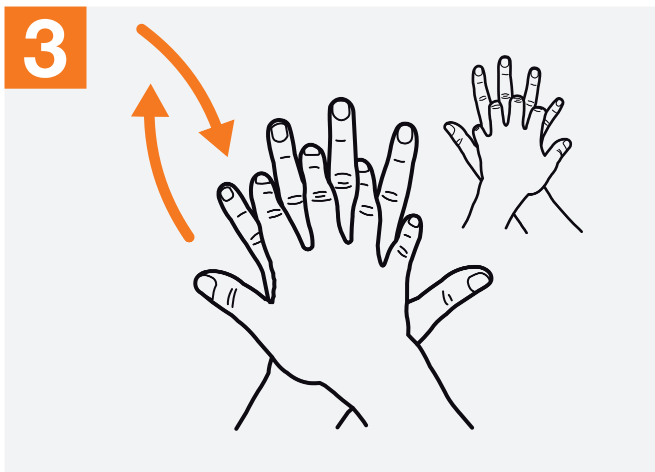

Right palm over left dorsum with interlaced fingers and vice versa;

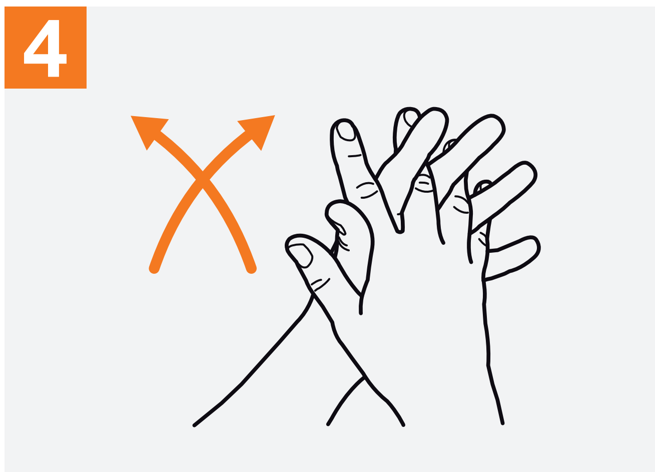

Palm to palm with fingers interlaced;

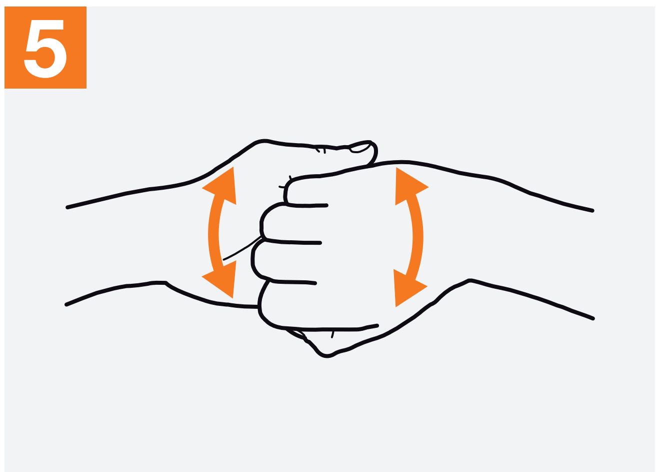

Backs of fingers to opposing palms with fingers interlocked;

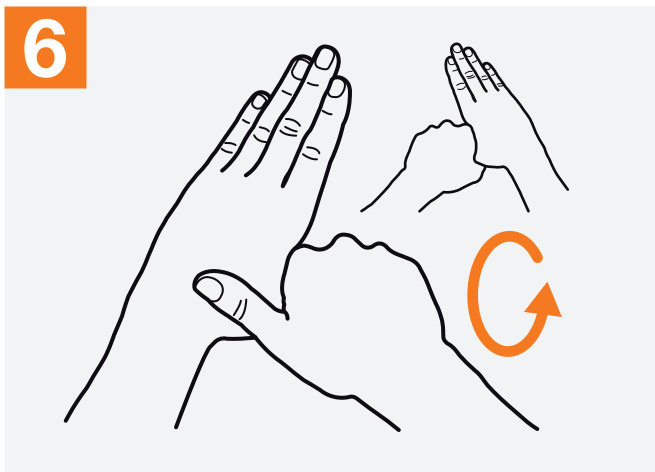

Rotational rubbing of left thumb clasped in right palm and vice versa;

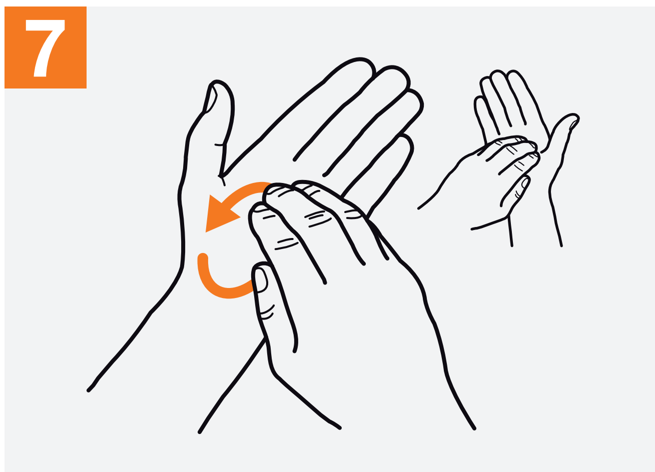

Rotational rubbing, backwards and forwards with clasped fingers of right hand in left palm and vice versa;

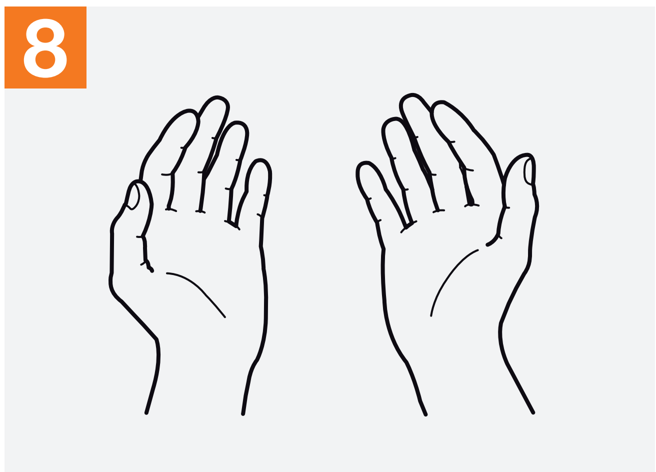

Once dry, your hands are safe.

World Health Organization

Patient Safety
A World Alliance for Safer Health Care

SAVE LIVES
Clean **Your** Hands

All reasonable precautions have been taken by the World Health Organization to verify the information contained in this document. However, the published material is being distributed without warranty of any kind, either expressed or implied. The responsibility for the interpretation and use of the material lies with the reader. In no event shall the World Health Organization be liable for damages arising from its use.

WHO acknowledges the Hôpitaux Universitaires de Genève (HUG), in particular the members of the Infection Control Programme, for their active participation in developing this material.

May 2009
