# Supplementary figures and images for: Accurate Measurement of Handwash Quality Using Sensor Armbands: Instrument Validation Study
Source: JMIR Mhealth Uhealth. 2020 Mar 26;8(3):e17001. doi: 10.2196/17001 (PMC7146248; doi:10.2196/17001)

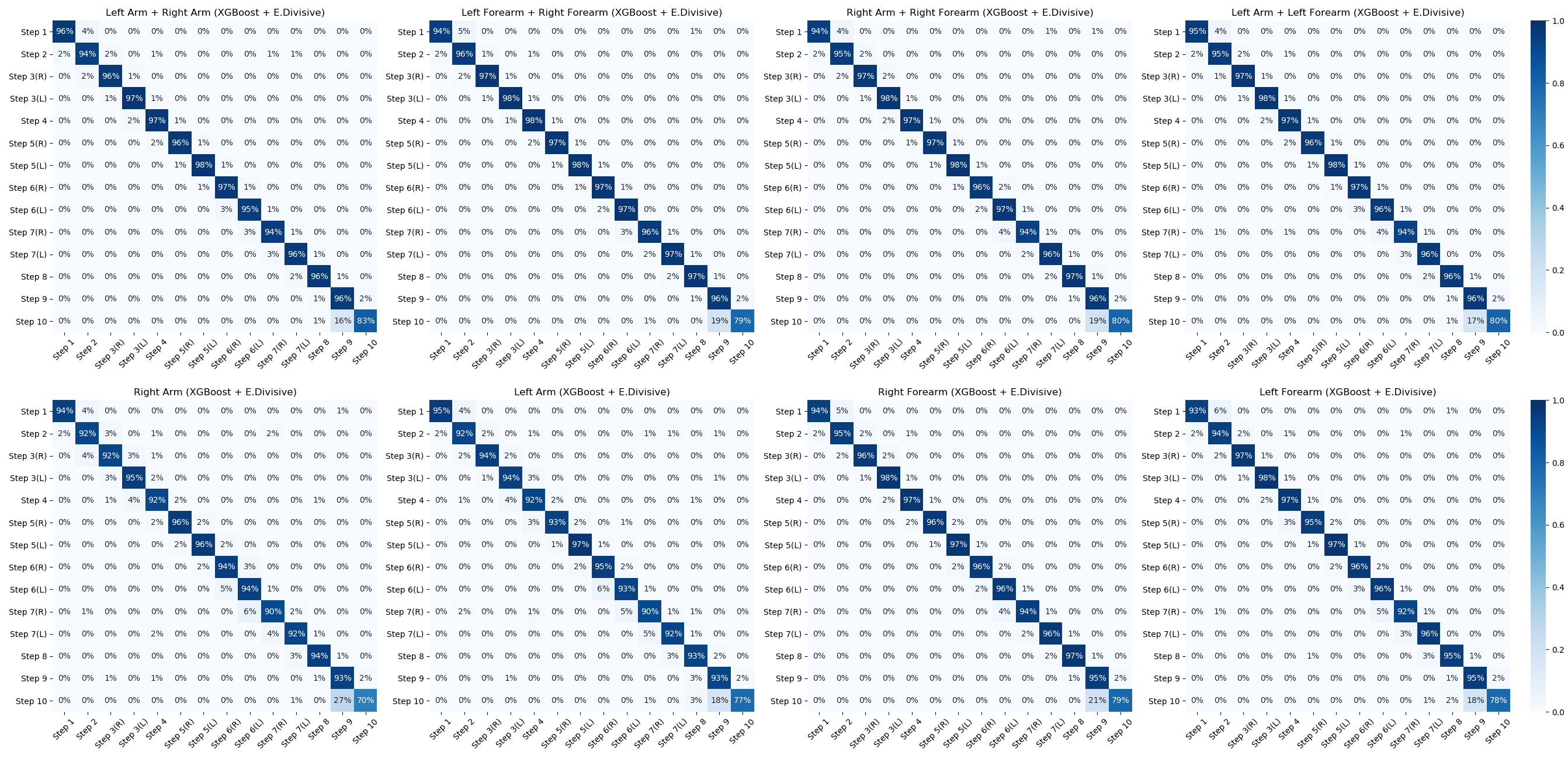

Supplement: Multimedia Appendix 4 [file mhealth_v8i3e17001_app4.png]

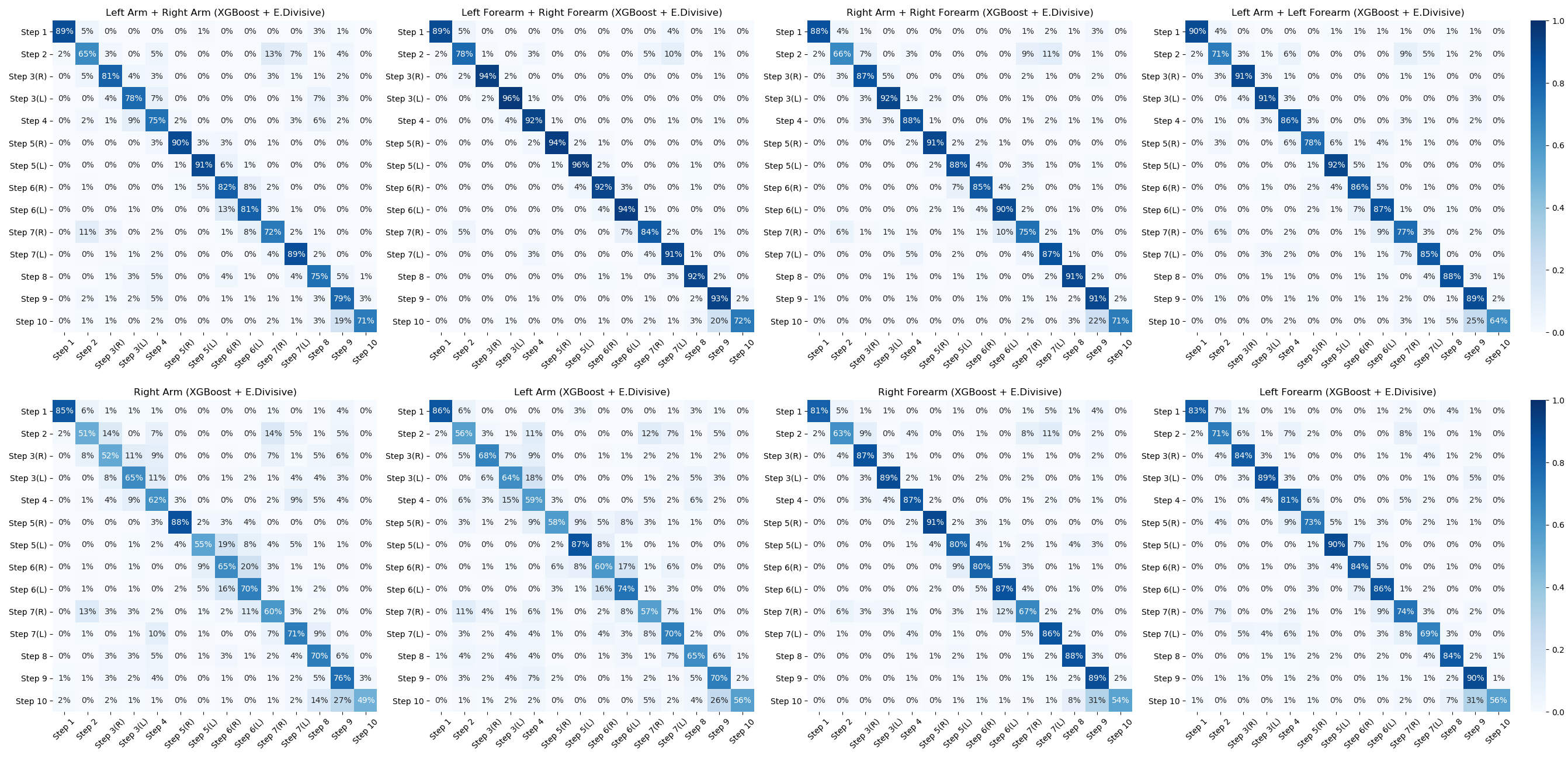

Supplement: Multimedia Appendix 5 [file mhealth_v8i3e17001_app5.png]
